# T63. TOWARDS A COMPREHENSIVE SEMANTIC MEMORY NETWORK IN SCHIZOPHRENIA: PRELIMINARY RESULTS USING MAGNETOENCEPHALOGRAPHY (MEG) IN SCHIZOTYPY

**DOI:** 10.1093/schbul/sby016.339

**Published:** 2018-04-01

**Authors:** Rachel Batty, Will Woods, Susan Rossell

**Affiliations:** 1 Swinburne University of Technology; 2 Swinburne University, Monash Alfred Psychiatry Research Centre

## Abstract

**Background:**

Semantic memory (memory for facts, concepts, and knowledge of the external world) abnormalities are predicted to underlie disturbances in thought and language, deficits in cognitive domains, and the development and maintenance of delusions in patients with schizophrenia. Electroencephalographic (EEG) recordings have successfully identified the neural time course for the processing of semantic information as an electrophysiological response between 300 and 500ms post stimulus (i.e., the N400). The N400 is a remarkably consistent and highly sensitive neural response to semantic relationships, and is thought to index the binding of current stimuli into context by detecting whether meaning is shared with recently processed stimuli or items in memory. To date, the N400 appears to be amodal: an index of semantic processing irrespective of stimulus type (e.g., word/picture stimuli alike), and has shown mixed findings in schizophrenia. However, existing literature has largely relied on EEG or functional magnetic resonance imaging (fMRI) techniques, and these are constrained in spatial and temporal resolution, respectively. Comparatively, MEG provides excellent spatio-temporal resolution, not possible from other stand-alone neuroimaging techniques. We aimed to determine the neuromagnetic correlates of novel semantic triads in both lexical and picture form, and to determine N400m differences in high/low schizotypal samples.

**Methods:**

MEG was recorded (whole-head 306 channel Elekta Neuromag® TRIUX magnetometer system) in 35 nonclinical controls (18 male) while completing a novel explicit semantic association task. MEG data were continuously sampled at 1KHz (0.1Hz high pass filter). Following MaxFiltering, data was processed using MNE for Python. Data were filtered offline (40Hz lowpass) and epoched at -300ms to 800ms post- target stimulus onset. The largest peak was measured at sensor triplets at temporo-parietal sites in both hemispheres. High/low schizotypal samples were determined by a median split of the Oxford-Liverpool Inventory of Feelings and Experiences (cognitive disorganisation scale; high=17, low=18).

**Results:**

Preliminary sensor level analyses demonstrated an N400m at temporo-parietal sites in response to both word and picture stimulus sets (with an earlier peak to pictures). Neither amplitude nor latency was significantly different between schizotypal samples, however a significant task x hemisphere x group interaction was found for N400m latency, F(1.00,33.00) = 6.18, p<.02.

**Discussion:**

An N400m was confirmed in response to the novel lexical task. The earlier peak (~200ms) to picture stimuli suggests that pictorial semantic information may be processed more rapidly than lexical information. The significant schizotypal group latency interaction demonstrated that while individuals low in schizotypal traits process lexical stimuli first in the right hemisphere (followed by the left) and picture stimuli first in the left hemisphere (followed by the right), individuals high in schizotypal traits do not demonstrate hemispheric specificity/laterality according to stimulus type. The data is currently being analysed for (i) source localisation, (ii) deep source contributions (e.g., hippocampus), and (iii) de/synchronisation of neural oscillations (across six frequency bands; 1-8Hz, 8-30Hz, 30-50Hz, 70-120Hz, 120-200Hz, and 200-300Hz).

